# Tumor Stroma Area and Other Prognostic Factors in Pancreatic Ductal Adenocarcinoma Patients Submitted to Surgery

**DOI:** 10.3390/diagnostics13040655

**Published:** 2023-02-09

**Authors:** Maria João Amaral, Mariana Amaral, João Freitas, Rui Caetano Oliveira, Marco Serôdio, Maria Augusta Cipriano, José Guilherme Tralhão

**Affiliations:** 1 General Surgey Department, Centro Hospitalar e Universitário de Coimbra, 3000-075 Coimbra, Portugal; 2 Faculty of Medicine, Universiy of Coimbra, 3000-548 Coimbra, Portugal; 3 Pathology Department, Centro Hospitalar e Universitário de Coimbra, 3000-075 Coimbra, Portugal; 4 Clinical Academic Center of Coimbra (CACC), 3000-075 Coimbra, Portugal; 5 Coimbra Institute for Clinical and Biomedical Research (iCBR) Area of Environment, Genetics and Oncobiology (CIMAGO), Faculty of Medicine, University of Coimbra, 3000-548 Coimbra, Portugal; 6 Biophysics Institute, Faculty of Medicine, University of Coimbra, 3000-548 Coimbra, Portugal

**Keywords:** pancreatic cancer, tumor microenvironment, stroma, prognosis, survival

## Abstract

Pancreatic ductal adenocarcinoma (PDAC) has a dense stroma, responsible for up to 80% of its volume. The amount of stroma can be associated with prognosis, although there are discrepancies regarding its concrete impact. The aim of this work was to study prognostic factors for PDAC patients submitted to surgery, including the prognostic impact of the tumor stroma area (TSA). A retrospective study with PDAC patients submitted for surgical resection was conducted. The TSA was calculated using QuPath-0.2.3 software. Arterial hypertension, diabetes mellitus, and surgical complications Clavien–Dindo>IIIa are independent risk factors for mortality in PDAC patients submitted to surgery. Regarding TSA, using >1.9 × 10^11^ µ^2^ as cut-off value for all stages, patients seem to have longer overall survival (OS) (31 vs. 21 months, *p* = 0.495). For stage II, a TSA > 2 × 10^11^ µ^2^ was significantly associated with an R0 resection (*p* = 0.037). For stage III patients, a TSA > 1.9 × 10^11^ µ^2^ was significantly associated with a lower histological grade (*p* = 0.031), and a TSA > 2E + 11 µ^2^ was significantly associated with a preoperative AP ≥ 120 U/L (*p* = 0.009) and a lower preoperative AST (≤35 U/L) (*p* = 0.004). Patients with PDAC undergoing surgical resection with preoperative CA19.9 > 500 U/L and AST ≥ 100 U/L have an independent higher risk of recurrence. Tumor stroma could have a protective effect in these patients. A larger TSA is associated with an R0 resection in stage II patients and a lower histological grade in stage III patients, which may contribute to a longer OS.

## 1. Introduction

Pancreatic ductal adenocarcinoma (PDAC) is the most common exocrine pancreatic neoplasm [1,2], representing the seventh leading cause of cancer-related deaths worldwide [1,2,3]. The five-year overall survival (OS) is only 10%, [4] which may be due not only to an aggressive and still poorly understood tumor biology, but also to a late diagnosis [5,6]. Patients with PDAC are often asymptomatic in the early stages of the disease [2,5,7] and due to the lack of appropriate early diagnostic markers, [7] only 15 to 20% of patients are diagnosed with resectable disease. Even for these patients, prognosis remains poor, although surgical resection remains the only treatment with curative potential [4].

Clinical and histopathological factors can have an impact on the OS and disease-free survival (DFS) of PDAC patients. Ensuring a complete and individualized assessment of the prognosis can allow patient stratification and obtain a more realistic estimate of OS and the potential of relapse [8]. The properties of the tumor microenvironment can be a potential prognostic resource [9]. PDAC is characterized by a dense stroma [10,11,12,13] that includes fibroblasts, immune cells, vasculature cells, and an extracellular matrix [14,15,16,17]. This stromal component, responsible for up to 50–80% of the tumor’s volume, [10,13,15] is important for understanding its cellular heterogeneity and the interactions that shape its architecture. According to this principle, the amount of stroma can be associated with the prognosis and resistance to therapy of these patients, although there are some discrepancies regarding its concrete impact [13]. In some studies, the amount of tumor stroma is identified as a factor contributing to immune suppression, biological aggressiveness, and tumor growth [10,11,15,16,18,19]. In other studies, a tumor suppressor role of PDAC-associated fibroblasts is revealed, indicating that stroma can also act as a barrier against the progression and development of metastases, [14,20] with fibrosis correlating with greater survival. However, this physical barrier can prevent penetration of chemotherapy drugs into the peri- and intratumoral environment, conferring resistance to therapy [15,16,17,18].

The aim of this work was to identify clinicopathological prognostic factors for PDAC patients submitted for surgical resection, and to study the prognostic impact of the tumor stroma area (TSA).

## 2. Materials and Methods

### 2.1. Study Design

This clinical retrospective study included 148 consecutive patients who underwent surgical resection for PDAC from March 2008 to December 2020, in our department, without neoadjuvant treatment. Routinely, a laparotomic cephalic pancreaticoduodenectomy (CPD) without pyloric preservation was the technique used for tumors with a cephalic location. Clinicopathological data were obtained by reviewing the patients’ clinical histories, using the hospital database records. The study was approved by our hospital’s Ethics Committee [21].

For preoperative analytical parameters, hyponatremia was considered when sodium <135 mEq/L, increased aspartate aminotransferase (AST) ≥35 U/L, increased alanine aminotransferase (ALT) ≥45 U/L, increased alkaline phosphatase (AP) >120 U/L, increased gamma-glutamyltransferase (GGT) ≥55 U/L and hypoalbuminemia when albumin <3.5 g/dL. For carbohydrate antigen (CA) 19.9 and carcinoembryonic antigen (CEA), cutoff values of 500 U/mL [22] and 5 ng/mL [23] were defined, respectively. Jaundice was considered for a total bilirubin (BR) value greater than 2.5 mg/dL [24]. Postoperative complications were defined as those occurring in the first 30 days after surgery and classified according to the Clavien–Dindo classification [25]. Delayed gastric emptying (DGE) [26] and postoperative pancreatic fistula (POPF) [27] were defined according to the International Study Group of Pancreatic Surgery. R1 resections were defined as those with a tumor-free margin ≤1 mm.

Lymph node (LN) ratio was calculated by dividing the number of invaded LNs by the number of resected LNs. The TSA was calculated for 78 patients. For each patient, 3 representative histological areas of the surgical specimen were selected by an expert pathologist and photographed with a total magnification of 20×. Image collection and analysis were blinded to the outcome. Hematoxylin and eosin-stained slides were observed using a light microscope (Nikon Eclipse 50i) and images were obtained using a Nikon Digital Slight DS-Fi1 camera. Subsequently, using the software [28,29] QuPath-0.2.3, the stromal area was delimited and calculated to then determine the mean of the 3 areas. Several cutoff values were tested for the study of the TSA. The DFS was calculated from the date of surgery to the date of relapse and the OS from the date of surgery to the date of death or of data analysis.

### 2.2. Statistical Analysis

Statistical analysis was performed using IMB SPSS software version 27.0 (IMB corporation, Armonk, NY, USA). Initially, a descriptive analysis of the results was undertaken. Metric variables were described by mean whenever there was a normal distribution of the values and by median if not. Relational statistics were conducted using the Fisher exact test and t Student test. Receiver operating characteristic (ROC) curves were used for TSA and LN ratios. Survival analysis was conducted using the Kaplan–Meier method and the corresponding log-rank tests. Univariate Cox regression was undertaken with the statistically significant variables from the survival analysis to identify predictors of mortality. Later, multivariate Cox regression was conducted. Hazard ratios (HR) and corresponding 95% confidence intervals (CI) and *p* values were reported. In all the tests used for statistical analysis, a *p*-value ≤ 0.05 was considered statistically significant.

## 3. Results

### 3.1. Clinicopathological Characteristics

A total of 93 patients (62.8%) were male and the median age at diagnosis was 70 years [interquartile range (IQR) 61–76]. Regarding patients’ comorbidities, 88 (59.5%) had arterial hypertension (AHT), 55 (37.2%) dyslipidemia, and 55 (37.2%) type 2 diabetes mellitus (DM), of which 28 (18.9%) were under metformin. As for preoperative symptoms and signs, 64 patients (43.2%) had abdominal pain and 93 (62.8%) had jaundice. The median Charlson comorbidity index was five (IQR 4–6), equivalent to a 21.4% estimated 10-year survival. Medians for preoperative sodium, AST, ALT, AP, GGT, total and direct BR, albumin, CA 19.9, and CEA are specified in Supplementary Materials. Prior to surgical intervention, 35 patients had hyponatremia, 99 (66.9%) increased AST and ALT, 111 (75%) increased GGT, and 48 (32.4%) hypoalbuminemia. CA 19.9 > 500 U/mL and CEA > 5 ng/mL were found in 44 (29.7%) and 35 (23.6%) patients, respectively.

The pancreatic head was the most frequent tumor location [126 patients (85.1%)] and CPD the most frequent surgical procedure [124 patients (83.8%)]. A blood transfusion was required in 49 patients (33.1%) (see Supplementary Materials). A total of 44 patients (29.7%) showed evidence of POPF and 85 (57.4%) had DGE. Supplementary Materials shows in detail the frequency observed in each degree of complication. A total of 58 patients (39.2%) did not receive adjuvant treatment, 46 (31.1%) underwent adjuvant chemotherapy, and 40 (27.0%) underwent adjuvant chemoradiotherapy.

Regarding histopathology, most patients were stage II [77 (52.0%)] and most tumors were classified as T2 [97 patients (65.5%)]. R0/R1 resection rates were 49.3% and 44.6%, respectively. Only 35 patients (23.6%) did not have lymph node invasion. Perineural invasion (PNI) was present in 133 cases (89.9%) and lymphovascular invasion (LVI) in 117 (79.1%). A total of 76 tumors (51.4%) were moderately differentiated. All results are detailed in Supplementary Materials.

**Figure 1:**
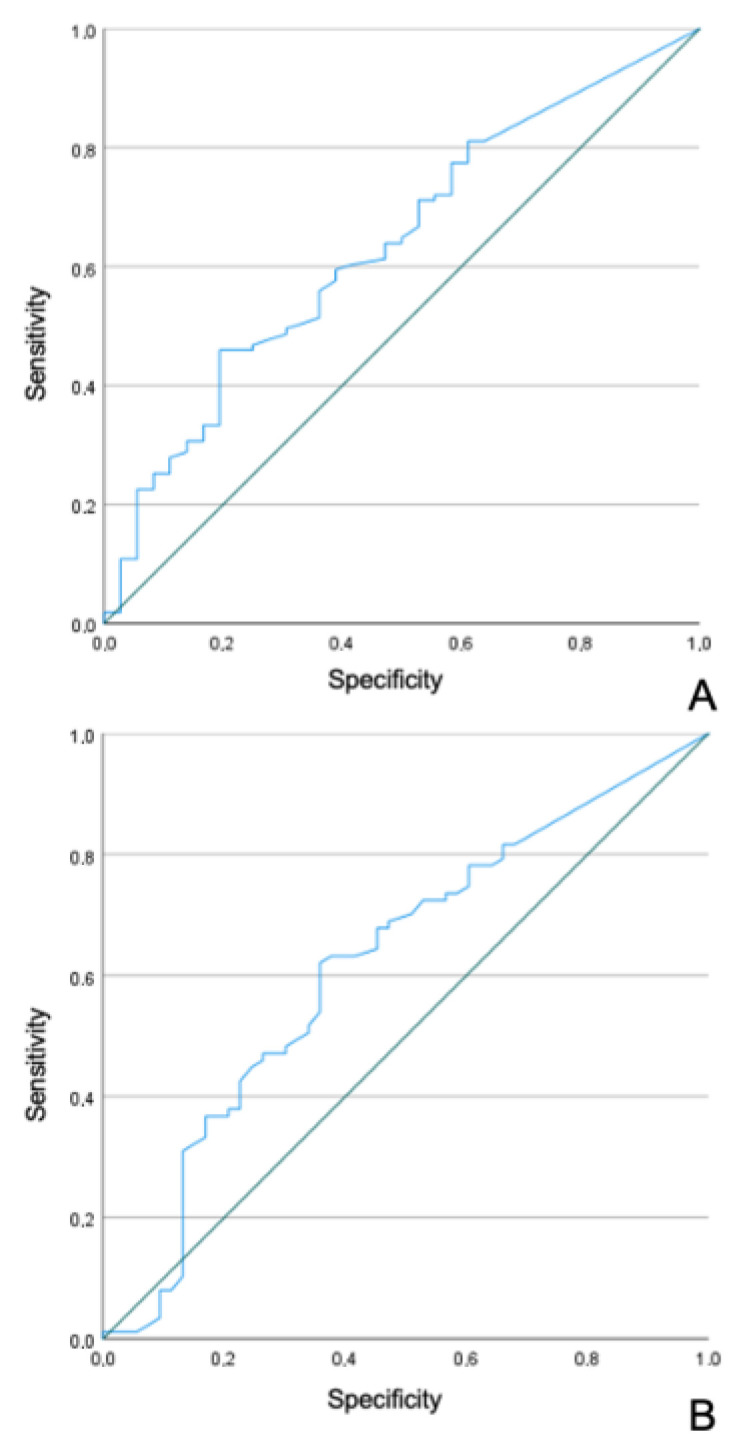
ROC cures for LN ratio and OS (**A**) and DFS (**B**).

Regarding the LN ratio, the analysis of the ROC curve obtained statistically significant results predicting OS (AUC 0.636, CI 95% 0.534–0.737, *p* = 0.015), with the best cutoff value at 0.009803922 (sensitivity 81.1%; specificity 36.1%) (Figure 1a). For DFS, results were also significant (AUC 0.620, CI 95% 0.522–0.717, *p* = 0.018), with the best cutoff value at 0.009803922 (sensitivity 81.6%; specificity 32.1%) (Figure 1b).

### 3.2. Follow-Up and Survival

The median follow-up time was 16.5 months (IQR 9.0–29.8). A total of 87 patients (58.8%) had a recurrence, of which 4.7% were local, 30.4% distant, and 22.3% local and distant recurrence. The liver was the most frequent site of recurrence (34.6%), followed by the lung (12.8%), the peritoneum (9%), and bone (1.3%). A total of 42.3% of patients had more than one site of recurrence. Median DFS was 13 months, the three-year DFS rate was 2.4%, and the five-year DFS rate was 1.2%. Median OS was 18 months and the OS survival rate at three and five years was 26.9% and 16.4%, respectively.

### 3.3. Tumor Stroma Area

The TSA mean was 1.718 × 10^11^µm^2^ (min-max 9.6 × 10^10^–2.3 × 10^11^). For all stages combined, using >1.9 × 10^11^ µm^2^ as a cutoff value, no statistically significant relation was found between stromal area and OS, although patients with higher TSAs seemed to have a longer OS (31 vs. 21 months, *p* = 0.495).

For stage II, patients with a TSA >1.9 × 10^11^ µm^2^ (21 vs. not reached, *p* = 0.072) and >2 × 10^11^ µm^2^ (21 vs. not reached, *p* = 0.099) seemed to have a higher OS, and a TSA > 2 × 10^11^ µm^2^ was significantly associated with an R0 resection (*p* = 0.037). In all patients with a TSA >2 × 10^11^ µm^2^, the resection margin was R0, and all had a CEA < 5 ng/mL, although this relationship was not statistically significant (*p* = 0.057). For stage III patients, a TSA > 1.9 × 10^11^ µm^2^ was significantly associated with a lower histological grade (G1) (*p* = 0.031), and a TSA > 2 × 10^11^ µm^2^ was significantly associated with a preoperative AP ≥ 120 U/L (*p* = 0.009) and a lower preoperative AST (≤35 U/L) (*p* = 0.004).

**Figure 2:**
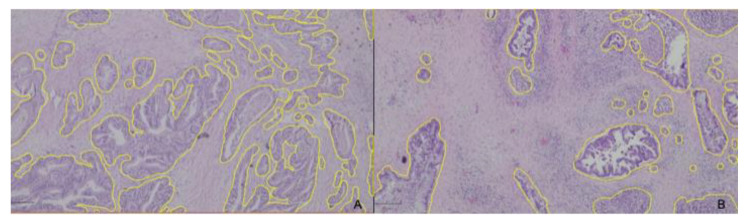
PDAC with a lower TSA (**A**) and a higher TSA (**B**). Yellow lines were used to determine the TSA by subtracting the glandular area from the total area, using the software QuPath-0.2.3.

Figure 2a,b shows histological images of PDAC, where the glandular areas were delimited. The remaining image corresponds to the tumor stroma.

The ROC curve analysis did not show statistically significant results for TSA, so it was not possible to identify cutoff values to predict OS [area under the curve (AUC) 0.544; CI 95% 0.407–0.680; *p* = 0.518] and DFS (AUC 0.490, CI 95% 0.359–0.621, *p* = 0.884).

### 3.4. Prognostic Factors

In the survival analysis, AHT, type 2 DM, abdominal pain, CA 19.9 > 500 U/mL, Clavien–Dindo > IIIa, stage > II, T > 2, lymph node invasion (N+), existence of metastases (M1), ILV, resection margin ≥ R1, tumor size > 2 cm, recurrence, and adjuvant treatment were correlated with OS (*p* < 0.05) (Table 1). Data regarding variables without statistically significant results are provided in Supplementary Materials.

As shown in Table 2, AHT, type 2 DM, abdominal, pain, CA 19.9 > 500 U/mL, T > 2, N+, M1, LVI, Clavien–Dindo > IIIa, stage > II, R ≥ 1, tumor size > 2 cm, and recurrence were significantly associated with worse OS in univariate regression. Adjuvant treatment was associated with a 38.2% reduction in the risk of death. Multivariate analysis showed that AHT, DM, and Clavien–Dindo > IIIa are independent risk factors for death in these patients (Table 2).

Preoperative serum levels of AST ≥35 U/L and ≥100 U/L, GGT ≥ 220 U/L and CA 19.9 > 500 U/mL were correlated with DFS (*p* < 0.05) (Table 3). Data regarding variables without statistically significant results are provided in Supplementary Materials. In univariate analysis, only AST ≥ 100 U/L and CA 19.9 > 500 U/mL were statistically significant risk factors for recurrence with an HR of 1.923 [CI 95% (1.197–3.088), *p* = 0.007] and of 1.672 [CI 95% (1.022–2.737), *p* = 0.041], respectively. Multivariate analysis showed that AST ≥ 100 U/L and CA 19.9 > 500 U/mL are independent risk factors for relapse, with an HR of 2.250 [CI 95% (1.317–3.842), *p* = 0.003] for AST and an HR of 2.250 [CI 95% (1.431–4.077), *p* = 0.001] for CA 19.9. GGT ≥ 220 U/L was not statistically significant [HR 0.901, (CI 95% 0.467–1.736), *p* = 0.755].

## 4. Discussion

One of the unique properties of pancreatic cancer is its excessive desmoplastic reaction, the deposition of an extensive extracellular matrix. Based on the results of our study and our methodology, tumor stroma could have a protective effect in PDAC patients, as patients with higher TSA seem to have longer OS, although without statistical significance. In addition, for stage III patients, a higher TSA had a significant relationship with a lower histological grade. A study demonstrated that PDAC is restrained by the stroma matrix [17]. Results from orthotopic animal models suggest that any hypothetical benefit afforded by possible improved drug availability following stromal matrix depletion is outweighed by protumorigenic effects on the tumor itself. Furthermore, patients with a higher tumor stromal density (TSD) had significantly longer OS than those with low TSD. Moreover, Rhim et al. demonstrated that the depletion of stromal cells in these tumors, targeting the Sonic hedgehog (Shh) pathway, resulted in poorly differentiated histology, increased vascularity and proliferation, and reduced survival in a pancreatic cancer mouse model [30]. Later, Nishida et al. showed that patients with low TSA harbored more poorly differentiated carcinomas, having a poorer prognosis [31]. They defined TSA as the ratio of the TSA to that of the total tumor area of 50x field. This supports the perspective that the stromal response might be a host response to inhibit tumor growth [32].

In stage III patients, a TSA >2 × 10^11^ µ^2^ was significantly associated with a preoperative AP ≥ 120 U/L, which could help predict the amount of tumoral stroma. Possibly, a higher TSA represents a higher tumor density and more obstruction to the bile duct. It has also been previously shown that pancreatic carcinoma secretes AP into the blood, although its elevation was associated with poor DFS and OS in these patients [33]. A study by Son et al. showed that PDAC cell growth relied on an AST dependent pathway, as knockdown of this transaminase significantly impaired PDAC growth in multiple cell lines and primary PDAC cells [34]. In our study, a TSA > 2 × 10^11^ µ^2^ was also significantly associated with a low AST in stage III patients.

In stage II PDAC patients, higher TSA was significantly associated with an R0 resection, which agrees with a study by Li et al. that revealed a R1 resection independently associated with a low stroma component [11]. Pancreatic resection with a positive margin was associated with poor survival and early recurrence [35] although many studies report a high rate of local recurrence not only in patients with R1 resections but also in cases of supposed R0 status [36]. A meta-analysis showed that adjuvant CT following pancreatic cancer resection improves OS, but no difference was obtained between R0 and R1 resections [37]. In our study, all patients with a TSA > 2 × 10^11^ µ^2^ had a CEA < 5 ng/mL, which could correlate with a better prognosis, as high serum CEA levels are associated with a poor prognosis in PDAC patients [38].

AHT, type 2 DM, abdominal pain, CA 19.9 > 500 U/mL, T > 2, N+, M1, LVI, Clavien–Dindo > IIIa, stage > II, R ≥ 1, tumor size > 2 cm, and recurrence were found as prognostic factors for OS in our analysis, which is in agreement with previous studies. Adjuvant treatment was associated with a reduction in the risk of death, prolonging OS, which was also previously known [39]. Multivariate analysis showed that AHT, DM, and Clavien–Dindo > IIIa are independent risk factors for mortality in these patients. This result seems to be shown for the first time for AHT. A metanalysis did not observe a statistically significant association between AHT and pancreatic cancer [40]. On the other hand, the association between DM and PDAC has been reported, the prevalence of PDAC is higher in adults with new-onset DM than in the general population [41], and patients with this comorbidity have higher mortality overall [42]. According to this, the potential beneficial role of metformin has been studied [43], but we did not obtain significant results for its effect on OS. Despite our results for AHT and DM, no significant difference was seen in AHT and DM rates across PDAC stages in the literature [41].

OS outcomes of patients undergoing surgical resection for PDAC have improved over the past two decades. The development of postoperative complications, mainly major complications, may delay or even preclude adjuvant treatment [44], which may justify the occurrence of major complications (Clavien–Dindo > IIIa) as a prognostic factor for these patients. In the univariate analysis of the study by Dhayat et al., Clavien–Dindo complications ≥IIIb and grade B and C pancreatic fistulas were associated with lower OS and DFS. In multivariate analysis, complications ≥IIIb were also associated with OS and DFS, but pancreatic fistula grade B and C were only associated with DFS. It is also thought that anastomotic leakage leads to inflammation with the release of pro-inflammatory cytokines that alter host defenses and promote growth of residual malignant cells [45].

Regarding the other prognostic factors in univariate analysis in our study, abdominal pain is usually associated with more advanced stages of PDAC, justifying its importance as predictor of outcome and survival. It entails neuropathic mechanisms due to neural infiltration by cancer cells. [46] CA 19.9 > 500 U/mL was associated with shorter OS and DFS and is already a well-established independent predictor of survival [22], having been tested with different cutoff values. Recurrence, as expected, was also associated with worse OS. In a recent observational study, additional treatment for PDAC recurrence was independently associated with OS, which shows that standardized postoperative surveillance aiming at early detection, before the onset of symptoms, has the potential to further improve survival [47].

As for histopathological factors (T > 2, N+, M1, LVI, R ≥ 1), we can say that longer OS can be achieved if PDAC is diagnosed in early stages. However, early diagnosis does not seem to prolong DFS as none of these factors proved to be associated with DFS. In other studies, a patient’s R-status was independently associated with long-term survival [44]. On the other hand, the number of positive lymph nodes is consistently associated with OS in most studies and evidence demonstrates that there is a negative association between OS and LNR in N1 patients [48,49]. LNR can therefore be an important tool, more so than the number of lymph nodes harvested [48], but the AUC we obtained was between 0.6 and 0.7 which indicates a poor ability of this factor to discriminate patients with better OS and DFS in our study.

In our cohort of patients, AST ≥ 100 U/L and CA 19.9 > 500 U/mL were independent risk factors for recurrence. In a study by Tian et al., a CA 19.9 ≥ 400 U/mL was also an independent risk factor for DFS [50]. Regarding AST, it has a role in PDAC cell growth, as previously mentioned. Furthermore, it has been shown that an elevation of pretreatment serum AST/ALT ratio predicts a poor disease outcome and response rate in patients with advanced PDAC treated with gemcitabine/nab-paclitaxel [51]. In other studies, adjuvant chemotherapy correlated with DFS and reduced the risk of recurrence [50,52], but this was not confirmed in our work.

There are some potential limitations in this study. First, it is a retrospective study from a single institution, with small sample size. Second, only 58.1% of patients received adjuvant treatment, so the tumor recurrence rate and DFS after surgery could be affected, although its administration complied with the protocols of the institution. However, up to 30% of PDAC patients do not receive adjuvant therapy because of the development of comorbidities, worsening of performance status, postoperative complications and/or early recurrence [39] and for those receiving the treatment, delays and dose modifications are common [53]. Third, evaluating only a small area of the tumor tissue may not adequately reflect its entire architecture, which could make this method insufficient to estimate prognosis. PDAC is also characterized by a stroma with different types of cells that can mediate malignant behavior, and this heterogeneity should be considered in further studies with detailed characterization.

In conclusion, patients with PDAC undergoing surgical resection and with a worse prognosis can be identified. AHT, DM, and surgical complications Clavien–Dindo > IIIa are independent prognostic factors for OS after surgery. Patients with preoperative CA 19.9 >500 U/L and AST ≥ 100 U/L have an independent higher risk of recurrence. Tumor stroma could have a protective effect in these patients. Larger stromal areas are associated with an R0 resection in stage II patients and a lower histological grade in stage III patients, which may contribute to longer OS.

## Figures and Tables

**Table 1:** Variables influencing overall survival. Abbreviations—CA 19.9: carbohydrate antigen 19.9; T: tumor.

	Mean OS (Months)	*p*
Arterial hypertension		
No	25	
Yes	16	0.01
Type 2 diabetes mellitus		
No	24	
Yes	14	0.003
Abdominal pain		
No	25	
Yes	15	0.008
CA 19.9		
≤500 U/mL	22	
>500 U/mL	15	0.022
Clavien–Dindo		
I, II, IIIa	20	
IIIb, IV, V	3	0.006
Stage		
I-II	21	
III-IV	14	0.001
T		
T1-2	21	
T3-4	16	0.009
Lymph nodes		
N0	24	
N+	17	0.01
Metastases		
M0	20	
M1	12	0.009
Lymphovascular invasion		
No	29	
Yes	17	0.005
Resection margin		
R0	22	
R1-2	17	0.014
Tumor size (cm)		
≤2	27	
>2	18	0.015
Adjuvant treatment		
No	14	
Yes	21	0.011
Recurrence		
No	65	
Yes	18	0

**Table 2:** Prognostic factors for overall survival in PDAC patients. Abbreviations—CA 19.9: carbohydrate antigen 19.9; HR: hazard ratio; M: metastasis; N: lymph nodes; R: resection margin; T: tumor. Overall survival calculated from the date of surgery to the date of death or of data analysis.

	Univariate Analysis		Multivariate Analysis	
	HR (CI 95%)	*p*	HR (CI 95%)	*p*
Arterial hypertension	1.641 (1.112–2.421)	0.013	1.912 (1.025–3.564)	0.041
Type 2 diabetes mellitus	1.759 (1.200–2.578)	0.004	2.554 (1.253–5.203)	0.010
Abdominal pain	1.647 (1.131–2.400)	0.009	1.364 (0.731–2.546)	0.329
CA 19.9 > 500 U/mL	1.638 (1.063–2.524)	0.025	1.410 (0.799–2.487)	0.236
Clavien–Dindo > IIIa	1.977 (1.198–3,263)	0.008	3.377 (1.365–8.352)	0.008
Stage > II	1.914 (1.288–2.844)	0.001	1.755 (0.872–3.534)	0.115
T > 2	1.703 (1.132–2.560)	0.011	1.112 (0.256–4.829)	0.888
N+	1.840 (1.144–2.959)	0.012	1.154 (0.539–2.470)	0.712
M1	2.030 (1.171–3.518)	0.012	1.230 (0.501–3.020)	0.651
Lymphovascular invasion	1.968 (1.202–3.223)	0.007	1.634 (0.741–3.601)	0.223
R ≥ 1	1.594 (1.091–2.331)	0.016	1.108 (0.594–2.068)	0.746
Tumor size > 2 cm	2.323 (1.148–4.700)	0.019	2.210 (0.612–7.977)	0.226
Adjuvant treatment	0.618 (0.422–0.906)	0.014	0.849 (0.388–1.860)	0.683
Recurrence	2.221 (1.399–3.524)	0.001	1.682 (0.762–3.715)	0.198

**Table 3:** Variables influencing disease-free survival. Abbreviations—AST: aspartate aminotransferase; ALT: alanine aminotransferase; GGT: gamma-glutamyl transferase; CA 19.9: carbohydrate antigen 19.9.

	Median DFS (Months)	*p*
AST		
Normal	12	
High (≥35 U/L)	9	0.04
AST		
<100 U/L	11	
≥100 U/L	6	0.004
GGT		
<220 U/L	12	
≥220 U/L	9	0.045
CA 19.9		
≤500 U/mL	12	
>500 U/mL	8	0.03

## Data Availability

All data generated or analyzed during this study are included in this published article (and its Supplementary Materials).

## Supplementary Materials

The following are available online at https://www.mdpi.com/article/10.3390/diagnostics13040655/s1, Table S1: Descriptive statistics of the study population, Table S2: Descriptive statistics of pathological characteristics, Table S3: Influence of other variables on overall survival, Table S4: Influence of other variables on disease-free survival.
