# Supplementary material for: Tumor Stroma Area and Other Prognostic Factors in Pancreatic Ductal Adenocarcinoma Patients Submitted to Surgery
Source: Diagnostics (Basel). 2023 Feb 9;13(4):655. doi: 10.3390/diagnostics13040655 (PMC9955223; doi:10.3390/diagnostics13040655)
Supplement: Supplementary file 1 [file diagnostics-13-00655-s001.zip › diagnostics-2209749-supplementary.pdf]

Supplementary Materials

Table S1 – Descriptive statistics of the study population.
IQR: interquartile range; AST: aspartate aminotransferase; ALT: alanine aminotransferase; AP: alkaline phosphatase; GGT: gamma-glutamyl transferase; BR: bilirubin; CA 19.9: carbohydrate antigen 19.9; CEA: carcinoembryonic antigen; CPD: cephalic pancreaticoduodenectomy; DDP: distal pancreatectomy; TDP: total pancreatectomy; CT: chemotherapy; RT: radiotherapy

**Variable**	**N=148**
**Age**	
Median (IQR)	70 (61-76)
Min-Max	40-93
**Sex**	
Male	93 (62.8%)
Female	55 (37.2%)
**Arterial hypertension**	88 (59.5%)
**Dyslipidemia**	55 (37.2%)
**Type 2 Diabetes mellitus**	55 (37.2%)
Metformin	28 (18.9%)
**Past history of other cancers**	17 (11.5%)
**Abdominal pain**	64 (43.2%)
**Jaundice**	93 (62.8%)
**Charlson comorbidity index**	
Median (IQR)	5 (4-6)
Min-Max	0-11
**Preoperative sodium** (mEq/L)	
Median (IQR)	137.0 (135.0-140.0)
Min-Max	124.0-144.7
**Preoperative AST** (U/L)	
Median (IQR)	60.0 (28.0-131.0)
Min-Max	9-668
**Preoperative ALT** (U/L)	
Median (IQR)	72.0 (31.0-170.5)
Min-Max	7-922
**Preoperative AP** (U/L)	
Median (IQR)	278.5 (118.5-458.0)
Min-Max	38-2100
**Preoperative GGT** (U/L)	
Median (IQR)	250.5 (69.3-635.8)
Min-Max	10-3001
**Preoperative total BR** (mg/dL)	
Median (IQR)	8.4 (1.0-17.2)
Min-Max	0.2-31.7
**Preoperative direct BR** (mg/dL)	
Median (IQR)	8.7 (2.5-14.1)
Min-Max	0.4-347.0
**Preoperative albumin** (g/dL)	
Median (IQR)	3.7 (3.3-4.2)
Min-Max	1.7-41.0
**Preoperative CA 19.9** (U/mL)	
Median (IQR)	225.8 (36.3-978.8)

Min-Max	2.0-23911.0
**Preoperative CEA** (ng/mL)	
Median (IQR)	3.3 (1.9-6.1)
Min-Max	0.6-179297.0
**Prolonged hospitalization** (≥17 days)	83 (56.1%)
**Type of Surgery**	
CPD	124 (83.8%)
DP	17 (11.5%)
TP	7 (4.7%)
**Clavien-Dindo**	
No complications	24 (16.2%)
I	21 (14.2%)
II	57 (38.5%)
IIIa	20 (13.5%)
IIIb	5 (3.4%)
IVa	2 (1.4%)
IVb	4 (2.7%)
V	13 (8.8%)
**Postoperative pancreatic fistula (POPF)**	
No	93 (62.8%)
BL	20 (13.5%)
B	12 (8.1%)
C	12 (8.1%)
**Delayed gastric emptying (DGE)**	
No	61 (41.2%)
A	44 (29.7%)
B	25 (16.9%)
C	16 (10.8%)
**Adjuvant treatment**	
No	58 (39.2%)
CT	46 (31.1%)
CT+RT	40 (27.0%)

Table S2 – Descriptive statistics of pathological characteristics.
IQR: interquartile range

Variable	N=148
**Stage**	
I	28 (18.9%)
II	77 (52.0%)
III	31 (20.9%)
IV	12 (8.1%)
**T**	
T1	14 (9.5%)
T2	97 (65.5%)
T3	37 (25.0%)
T4	0 (0%)
**N**	
N0	35 (23.6%)
N1	79 (53.4%)
N2	34 (23.0%)
**Metastases**	
M0	133 (89.9%)
M1	15 (10.1%)
**Resected lymph nodes**	
Median (IQR)	18 (13-25)
Min-Max	3-62
**Invaded lymph nodes**	
Median (IQR)	2 (1-3)
Min-Max	0-12
**Lymph nodes ratio**	
Median (IQR)	009 (0.03-0.19)
Min-Max	0.00-0.86
**Histological grade**	
Well differentiated (G1)	49 (33.1%)
Moderately differentiated (G2)	76 (51.4%)
Poorly differentiated (G3)	12 (8.1%)
**Perineural invasion**	133 (89.9%)
**Lymphovascular invasion**	117 (79.1%)
**Resection margin**	
R0	73 (49.3%)
R1	66 (44.6%)
R2	9 (6.1%)
**Tumor size** (cm)	
Median (IQR)	3,2 (2.5-4.0)
Min-Max	0.4-11.0

Table S3 – Influence of other variables on overall survival (OS).
OS: overall survival; AST: aspartate aminotransferase; ALT: alanine aminotransferase; AP: alkaline phosphatase; GGT: gamma-glutamyl transferase; CEA: carcinoembryonic antigen; CPD: cephalic pancreaticoduodenectomy; POPF: postoperative pancreatic fistula; DGE: Delayed gastric emptying; CT: chemotherapy; RT: radiotherapy

Variable	Median OS (months)	*p*
**Age**		
<70	18	
≥70	18	0.345
**Sex**		
Male	18	
Female	19	0.749
**Dyslipidemia**		
No	18	
Yes	17	0.698
**Metformin**		
No	16	
Yes	13	0.437
**Past history of other cancers**		
No	18	
Yes	18	0.337
**Jaundice**		
No	17	
Yes	20	0.075
**Preoperative hyponatremia** (<135 mEq/L)		
No	19	
Yes	16	0.430
**Preoperative AST**		
Normal	19	
High (≥35 U/L)	17	0.800
**Preoperative ALT**		
Normal	19	
High (≥45 U/L)	17	0.079
**Preoperative AP**		
Normal	20	
High (≥120 U/L)	17	0.956
**Preoperative GGT**		
Normal	21	
High (≥55 U/L)	17	0.382
**Preoperative hypoalbuminemia** (<3,5 g/dL)		
No	18	
Yes	17	0.632
**Preoperative CEA**		
≤5 ng/mL	21	
>5 ng/mL	17	0.764
**Type of surgery**		
CPD	18	
Others	18	0.333
**Surgery duration**		
<7h	20	
≥7h	18	0.711
**Blood transfusion**		
No	17	
Yes	19	0.851
**POPF**		
No	18	
Yes	20	0.788
**POPF grade**		

Biochemical leak	24	
Grade B e C	13	0.082
**DGE**		
Grade A	21	
Grade B ou C	15	0.414
**Histological grade**		
Well differentiated	19	
Moderately/poorly differentiated	17	0.203
**Perineural invasion**		
No	30	
Yes	17	0.906
**Type of adjuvant treatment**		
CT	21	
CT+RT	21	0.785
**Local recurrence**		
No	19	
Yes	18	0.124
**Distant recurrence**		
No	18	
Yes	17	0.390
**Local and distant recurrence**		
No	18	
Yes	19	0.899
**Pulmonary recurrence**		
No	19	
Yes	17	0.480
**Hepatic recurrence**		
No	20	
Yes	15	0.127
**Pulmonary and hepatic recurrence**		
No	18	
Yes	21	0.739
**Peritoneal recurrence**		
No	19	
Yes	18	0.280

Table S4 – Influence of other variables disease-free survival (DFS).
DFS: disease-free survival; AST: aspartate aminotransferase; ALT: alanine aminotransferase; AP: alkaline phosphatase; GGT: gamma-glutamyl transferase; CEA: carcinoembryonic antigen; CPD: cephalic pancreaticoduodenectomy; POPF: postoperative pancreatic fistula; DGE: delayed gastric emptying; CT: chemotherapy; RT: radiotherapy

Variable	Median DFS (months)	*p*
**Age**		
<70 years	10	
≥70 years	10	0.698
**Sex**		
Male	10	
Female	10	0.631
**Arterial hypertension**		
No	10	
Yes	10	0.331
**Dyslipidemia**		
No	10	
Yes	10	0.359
**Type 2 Diabetes mellitus**		
No	10	
Yes	9	0.244
**Metformin**		
No	10	
Yes	9	0.625
**Past history of other cancers**		
No	10	
Yes	7	0.537
**Abdominal pain**		
No	10	
Yes	10	0.140
**Jaundice**		
No	11	
Yes	10	0.851
**Preoperative hyponatremia** (<135 mEq/L)		
No	10	
Yes	9	0.345
**Preoperative ALT**		
Normal	12	
High (≥45 U/L)	9	0.229
**Preoperative AP**		
Normal	12	
High (≥120 U/L)	10	0.352
**Preoperative GGT**		
Normal	12	
High(≥55 U/L)	10	0.366
**Preoperative hypoalbuminemia** (<3,5 g/dL)		
No	10	
Yes	7	0.152
**Preoperative CEA**		
≤5 ng/mL	11	
>5 ng/mL	8	0.572
**Type of surgery**		
CPD	10	
Others	12	0.714
**Surgery duration**		
<7h	10	
≥7h	9	0.246
**Blood transfusion**		

No	9	
Yes	11	0.872
**Clavien-Dindo**		
I, II, IIIa	10	
IIIb, IV, V	6	0.500
**POPF**		
No	10	
Yes	8	0.345
**POPF grade**		
Biochemical leak	10	
Grade B e C	8	0.809
**DGE**		
Grade A	10	
Grade B ou C	6	0.442
**Stage**		
I-II	10	
III-IV	6	0.448
**T**		
T1-2	10	
T3-4	10	0.889
**Lymph nodes**		
N0	12	
N+	10	0.114
**Histological grade**		
Well differentiated	10	
Moderately/poorly differentiated	10	0.565
**Perineural invasion**		
No	12	
Yes	10	0.336
**Lymphovascular invasion**		
No	11	
Yes	10	0.723
**Resection margin**		
R0	11	
R1-2	8	0.119
**Tumor size** (cm)		
≤2	13	
>2	10	0.924
**Adjuvant treatment**		
No	8	
Yes	10	0.416
**Type of adjuvant treatment**		
CT	10	
CT+RT	10	0.999
